# Geographical Distribution of Scorpion *Odontobuthus doriae* in Isfahan Province, Central Iran

**Published:** 2017-09-08

**Authors:** Rouhullah Dehghani, Hamid Kassiri

**Affiliations:** 1 Social Determinants of Health (SDH) Research Center, Kashan University of Medical Sciences, Kashan, Iran; 2 Ahvaz Jundishapur University of Medical Sciences, School of Health, Ahvaz, Iran

**Keywords:** Bio-Ecology, Spatial distribution, *Odontobuthus doriae*, Iran

## Abstract

**Background:**

Scorpions are among the world’s venomous arthropods, they sting humans every year, suffering painful symptoms or losing their lives because of the venom. *Odontobuthus doriae* Thorell 1876 (Arachnida: Scorpionida: Buthidae) is a scorpion of medical importance and therefore its geographical distribution in Isfahan Province has been studied.

**Methods:**

This descriptive cross–sectional study was designed between Mar and Jun in 2012 and 2013 in Province of Isfahan, central Iran. Overall, 164 *O. doriae* scorpions were collected from their natural habitat by identifying the dug burrows. This arthropod’s burrows were identified based on the presence of tumuli, particularly between May and Jun at the sloping foothills of pristine embankments. The sampling data was categorized and compared.

**Results:**

The relative frequency of collected *O. doriae* for the counties was Mobarakeh (13.5%), Shahinshahre (11.5%), Borkhar (9%), Shahreza (7.5%), Kashan (7.5%), Naeen (6%), Natanz (5.5%), Isfahan (4.8%), Najafabad (4.8%), Aran and Bidgol (4.8%), Dehaghan (4.8%), Flavarjan (3.7%), Khomeinishahr (3.7%), Tiran (3.7%), Golpayegan (3.7%), Ardestan (3.7%) and Lenjan (2.5%). No *O. doriae* was collected from other counties of the province.

**Conclusion:**

The habitats of *O. doriae* are more often located in central, eastern and northern regions of the province compared to other regions. Counties of southern and western regions are among cold parts of the province.

## Introduction

Scorpions are among the oldest organisms on the earth. Scorpions possess stings equipped with venom glands located near the metasoma. Thus, they are among the most dangerous arthropods to humans. Approximately, 1.2 to 1.5 million cases of scorpion envenomation occurs worldwide annually (1, 2). Due to its type of climate, Iran hosts a very rich population of arthropods, and scorpions in particular (3, 5) and it is among the countries in which many scorpion species, particularly the dangerous types have been reported. Scorpion envenomation reports from ancient Iran are found in religious and historical texts, which indicate the long history of this issue in Iran (6, 7). Naturally, with the presence of these arthropods all over the Iranian territory, which possess suitable climate for their habitation, scorpion envenomation has been continuously an issue in Iran (8).

Scorpions are classified as burrowing and non-burrowing, in terms of digging burrows. Different scorpion species in the world dig burrows (9, 11). In Iran, *Odonthobuthus* spp. and *Scorpio maurus* dig burrows (12). Non-burrowing scorpions use natural and artificial materials as shelter. Non-burrowing scorpions, which enter human dwellings, too, take shelter in places like wardrobes, inside the shoes and boots, and under the objects laid on the ground. Other shelters of non-burrowing scorpions include inside the wall cracks, underneath the stones, camelthorn, small pieces of wood and leaves, and inside the gaps and cracks in the trees. Burrowing scorpions possess digging capabilities and are capable of digging 25 to 50cm deep burrows (13, 14). Humans may encounter burrowing scorpions infrequently under normal circumstances, but in emergency or military conditions, encounters with the burrowing scorpions are more likely due to excavations and digging trenches. Since most of the country is military bases and areas are located outside cities, and due to the distribution of dangerous types of scorpions, this issue is of great importance in terms of military health and hygiene. The scope of the presence of many species of this arthropod in military bases across the country and in isolated regions is such that soldiers constantly encounter them, and are envenomed by them. As such, these scorpions raise issues for military forces isolated from medical centers (15).

Scorpion envenomation is common in Middle Eastern countries, which is a significant issue in Iran’s southwest, in particular, posing many problems including allergic reactions (16, 18). Scorpion sting symptoms in humans include quickened breathing, paralysis of diaphragm, spasms of voluntary muscles, severe twitching, convulsions, muscle contraction and tension due to increased release of acetylcholine, pulmonary edema and swelling, damages to the heart muscle, vascular turbulence and disorders, impaired kidney function, necrosis and skin injuries and pathological changes in single or multiple organs. These symptoms vary depending on the sting and the impact mechanism of the venom of different species (19, 22).

One of the medically important scorpions is *O. doriae*, which exists in relatively high numbers in Iran. LD_50_, i.e. the lethal dose, of this arthropod’s venom is 0.19mg/kg for mice (8, 16) and thus studying it’s biological aspects is of value. Treatment and preventive measures are important issues in the field of medicine and hygiene. As such, examining other life aspects of scorpions, including *O. doriae*, such as their biology and ecology is of importance. Conducting such research in the field of bioecology is very time-consuming. Different studies, therefore, need to be unified to reach a conclusion and overcome the issues regarding scorpion envenomation.

## Materials and Methods

This descriptive cross–sectional study was designed between Mar and Jun in 2012 and 2013 in Province of Isfahan, central Iran. Overall, 164 *O. doriae* scorpions were collected from their natural habitats in different counties by locating their burrows. The burrows were subsequently excavated or filled with water.

To select the sampling area, a pristine piece of land of approximately 100m^2^, located 3 to 10km from the city being studied, was selected and all its identifiable burrows were searched to collect the arthropod. Identification of the burrows was based on two approaches (1). The first approach was based on the identification of burrows with asymmetric elliptical openings with minor axes of 1.2 to 1.5cm and major axes of 2.5 to 4.5cm, found in pristine or artificial embankments. In the second approach, the presence of tumuli, particularly between March and Jun at the sloping foothills of pristine embankments, was indicative of this arthropod’s presence. Tumuli at sloping foothills move to the lower side, which is indicative of fresh digging and clearing of the burrow. Excavation was carefully conducted using military shovels and trowels, and the arthropod was usually transferred to the sample case by forceps after full excavation of the burrow. In other cases, 1 to 3L of water was slowly poured into the identified burrows and the arthropod was caught with forceps upon exiting. Samples were transferred to laboratory for further examinations. The obtained data from the studied regions, regional characteristics, number of samples and catching methods were recorded at each sample and presented by illustrations and descriptive tables.

### The Studied Region

**Fig. 1.**
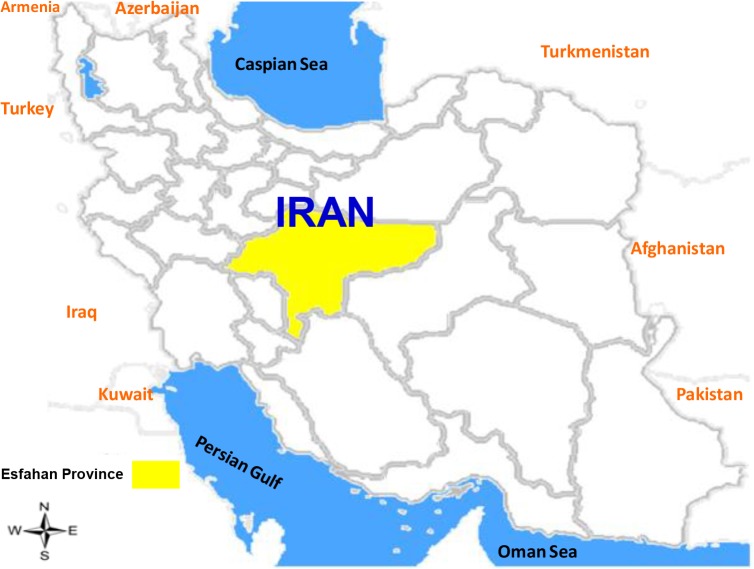
The geographical situation of Isfahan Province in Iran

Isfahan Province, with an area of 105937 square km, is situated in the center of Iran. It borders the provinces of Markazi, Qom, and Semnan to the north, Fars and Kohgiluyeh and Boyer-Ahmad to the south, Lorestan and Chaharmahal and Bakhtiari to the west, and Yazd and Khorasan to the east (Fig. 1). According to the latest administrative divisions, it has 23 counties, 60 cities, 37 districts, and 116 villages and its capital city is Isfahan. Counties of Isfahan Province include Isfahan, Ardestan, Borkhar, Shahreza, Khomeinishahr, Khvansar, Semirom, Fereydan, Fereydonshahre, Chadegan, Daran, Dehaghan, Kashan, Aran and Bidgol, Falavarjan, Golpayegan, Lenjan, Mobarakeh, Naeen, Najafabad, Tiran, Natanz and Shahinshahre. In general, the province has a moderate and dry climate. The west of the province, Isfahan County and western and southwestern regions of the province experience arid, semi-arid and cold semi-humid climates, respectively. The maximum, minimum, and average annual temperatures have been recorded as 40.6 °C, −10.6 °C and 16.7 °C, respectively, according to a report of the synoptic weather station in Isfahan. According to the same report, the number of the province’s frost days is 76, and the average annual rainfall is 116.9mm (23, 24).

## Results

**Fig. 2.**
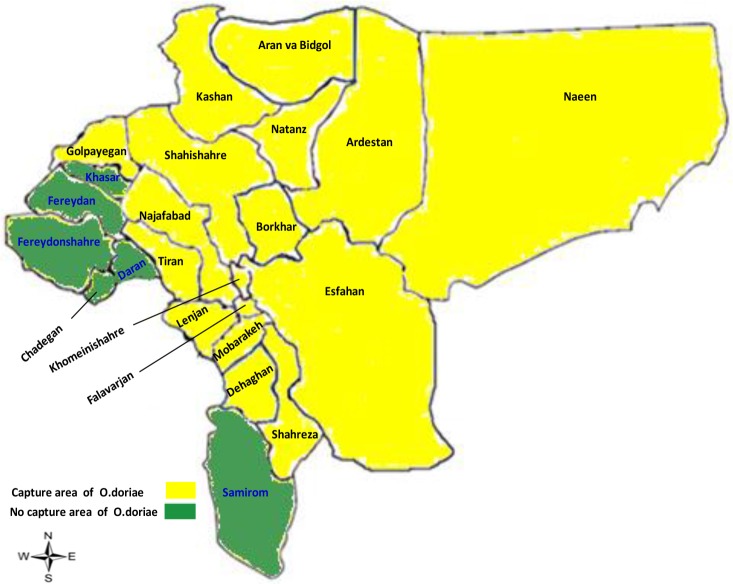
Study regions of *Odontobuthus doriae* in the Isfahan Province by county

The *O. doriae* scorpions were collected in 2012 and 2013 through 23 samplings. Overall, 164 scorpions of this species were collected. Characteristics of the collecting regions, collecting methods and number of the collected scorpions of the stated species are presented in Fig. 2 and Table 1. A minimum of 17 out of 23 counties had *O. doriae* scorpions. Maximum and minimum *O. doriae* catch were from Mobarakeh, at 13.5%, and Lenjan, at 2.5%, respectively. Moreover, 83% of the samples were collected by the excavation method and 17% were collected by pouring water into the burrows of *O. doriae*.

**Table 1.** *Odontobuthus doriae* abundance caught in Isfahan Province by county

**City**	**Methods of collecting**		**Number**	**Percent**

	**Digging**	**Use of water**		
**Mobarakeh**	15	7	22	13.5
**Shahinshahre**	17	2	19	11.5
**Borkhar**	15	-	15	9
**Shahreza**	12	-	12	7.5
**Kashan**	8	4	12	7.5
**Naeen**	6	4	10	6
**Natanz**	6	3	9	5.5
**Isfahan**	5	3	8	4.8
**Najafabad**	8	-	8	4.8
**Aran va Bidgol**	6	2	8	4.8
**Dehaghan**	8	-	8	4.8
**Flavarjan**	6	-	6	3.7
**Khomeinishahre**	6	-	6	3.7
**Tiran**	6	-	6	3.7
**Golpayegan**	6	-	6	3.7
**Ardestan**	2	3	5	3
**Lenjan**	4	-	4	2.5
**Daran**	-	-	-	-
**Chadegan**	-	-	-	-
**Khansar**	-	-	-	-
**Fereydan**	-	-	-	-
**Fereydonshahre**	-	-	-	-
**Semirom**	-	-	-	-
**23**	136	28	164	100

**Fig. 3.**
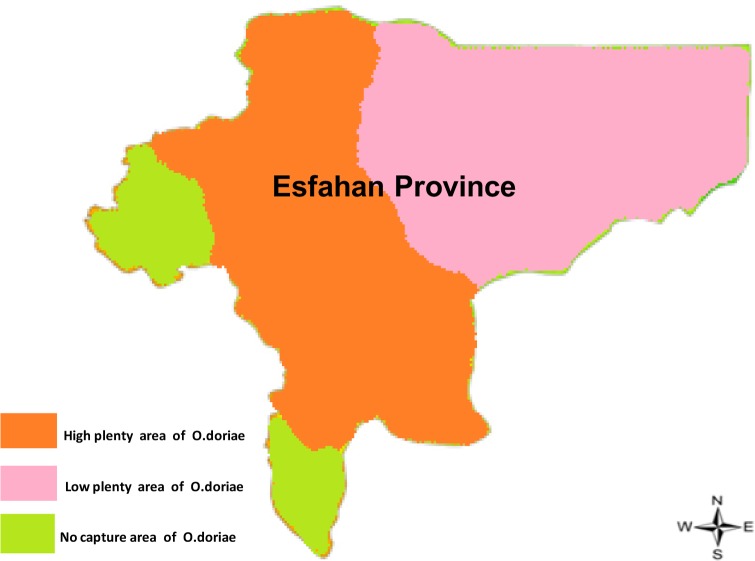
Study regions of in the Isfahan Province by catch abundance of *Odontobuthus doriae*

The highest percentage of the collected samples, after Mobarakeh, was from Shahinshahre, Borkhar, Shahreza, Kashan, Naeen, Natanz, Isfahan, Najafabad, Aran and Bidgol, Dehaghan, Flavarjan, Khomeini Shahr, Tiran, Golpayegan, Ardestan and Lenjan, at 11.5, 9, 7.5, 7.5, 6, 5.5, 4.8, 4.8, 4.8, 4.8, 3.7, 3.7, 3.7, 3.7, 3.7 and 2.5 percent, respectively. Meanwhile, no *O. doriae* scorpions were collected form Khansar, Semirom, Fereydan, Fereydonshahre, Chadegan and Daran in this study (Table 1). Most collected *O. doriae* was from the central regions of Isfahan Province, which includes the counties of Isfahan, Borkhar, Shahreza, Khomeini Shahr, Dahaghan, Kashan, Aran and Bidgol, Flavarjan, Golpayegan, Lenjan, Mobarakeh, Naeen, Najafabad, Tiran, Natanz, and Shahinshahre. The least *O. doriae* catch was from the eastern part of Isfahan Province, which includes northeastern Naeen, Northern Ardestan, Khor and Biabanak, and Anarak. No *O. doriae* scorpions were collected form Semirom, in the south of the province, Khansar, Fereydonshahre, Chadegan and Daran (Fig. 2, 3).

## Discussion

*Odontobuthus doriae* is a burrowing scorpion that is capable of digging burrows of up to 40cm. This species reaches up to 10cm in size. Coloration ranges from light yellow to dark and the body appendages are usually lighter than prosoma (25). This has been the predominant scorpion species of the studied regions in the natural habitats of Isfahan Province. Major signs of this arthropod’s burrows are the shape of the burrow’s opening and the presence of piled soil close to the burrow that is due to digging or clearing in early spring (13). These arthropods spend the winters in hibernation and are capable of restarting their regular functionality in favorable conditions within three to four hours (8), and freshly piled soil close to the burrow is indicative of this activity. At the opening of freshly dug burrows of this scorpion, certain type of tumulus can be observed, moved or washed away by wind or rain after a while. The shape of the burrow’s opening was distinct from other burrows and was of asymmetric elliptical shape with a minor axis of 1.2 to 1.5cm and a major axis of 2.5 to 4.5cm. While the opening resembles an ellipse, the burrow assumes a relatively circular shape after 2 to 3cm.

*Odontobuthus doriae* is of genus *Odontobuthus* and 3 species of it have been reported in Iran (26, 28). The species *O. doriae* has been reported in many regions of Iran, including the provinces of Yazd, Kerman (Kerman, Baft, Sirjan, Rafsanjan, Zarand, Shahrbabak, Kahnoj, Manojan, Shahdad), Markazi (Arak), Qazvin, Tehran (Deserts around Tehran County, Shemiran, Varamin), Alborz (Karaj), Semnan (Garmsar), West Azerbaijan (Uromieh, Nazlo, Salmas), Kermanshah, Bushehr (Borazjan), Hamadan, Hormozgan (Bandar Abbas) (29, 35), which corresponds with our study. Due to the remoteness of the habitats of this species of the human environment, its envenomation has been reported only sporadically (36). The maximum *O. doriae* hunting was from the central regions of Isfahan Province and the minimum *O. doriae* hunting was from the eastern part of Isfahan Province.

One of the major reasons for varying numbers of this arthropod’s burrows in Isfahan Province is due climatic diversity in this province. Semi-arid climate covers the central regions of Isfahan Province. Dryness and little precipitation are typical characteristics of this climate. However, Zayanderud River considerably affects the climate of this region in a positive way and makes it more temperate. The study suggested that fewer *O. doriae* scorpions were observed in eastern part of Isfahan Province than in the central regions of this province. Arid climate covers northern Naeen, Biabanak, and Anarak to northern Ardestan. Abrupt changes in temperature, little precipitation, and strong winds during the year are characteristic of this climate (2). No *O. doriae* scorpions were collected from south of the province and its eastern counties in our study. These regions have far colder climates than other regions of the province and are higher in elevation. Higher elevations and lower temperatures can lead to fewer favorable habitats for this arthropod. Extreme cold hinders the activities and spreading of scorpions (37).

## Conclusion

*Odontobuthus doriae* has more habitats in central, eastern and northern parts of the province than in other parts. Southern and western counties are among the cold regions of the province. Since scorpions occupy a wide range of habitats in warmer regions, conducting research on the correlation of the distribution of medically important species and different climate factors with scorpion envenomation can provide new insights into this issue.
